# Supplementary material for: Enhanced light-matter interaction in an atomically thin semiconductor coupled with dielectric nano-antennas
Source: Nat Commun. 2019 Nov 11;10:5119. doi: 10.1038/s41467-019-12963-3 (PMC6848120; doi:10.1038/s41467-019-12963-3)
Supplement: Supplementary file 2 — Supplementary Information [file 41467_2019_12963_MOESM2_ESM.pdf]

Supplementary Information for Enhanced light-matter interaction in an atomically thin semiconductor coupled with dielectric nano-antennas

L. Sortino et al.

SUPPLEMENTARY NOTE I: SPATIAL DISTRIBUTIONS OF ELECTRIC AND MAGNETIC FIELDS PRODUCED BY GALLIUM PHOSPHIDE DIMER NANO-ANTENNAS

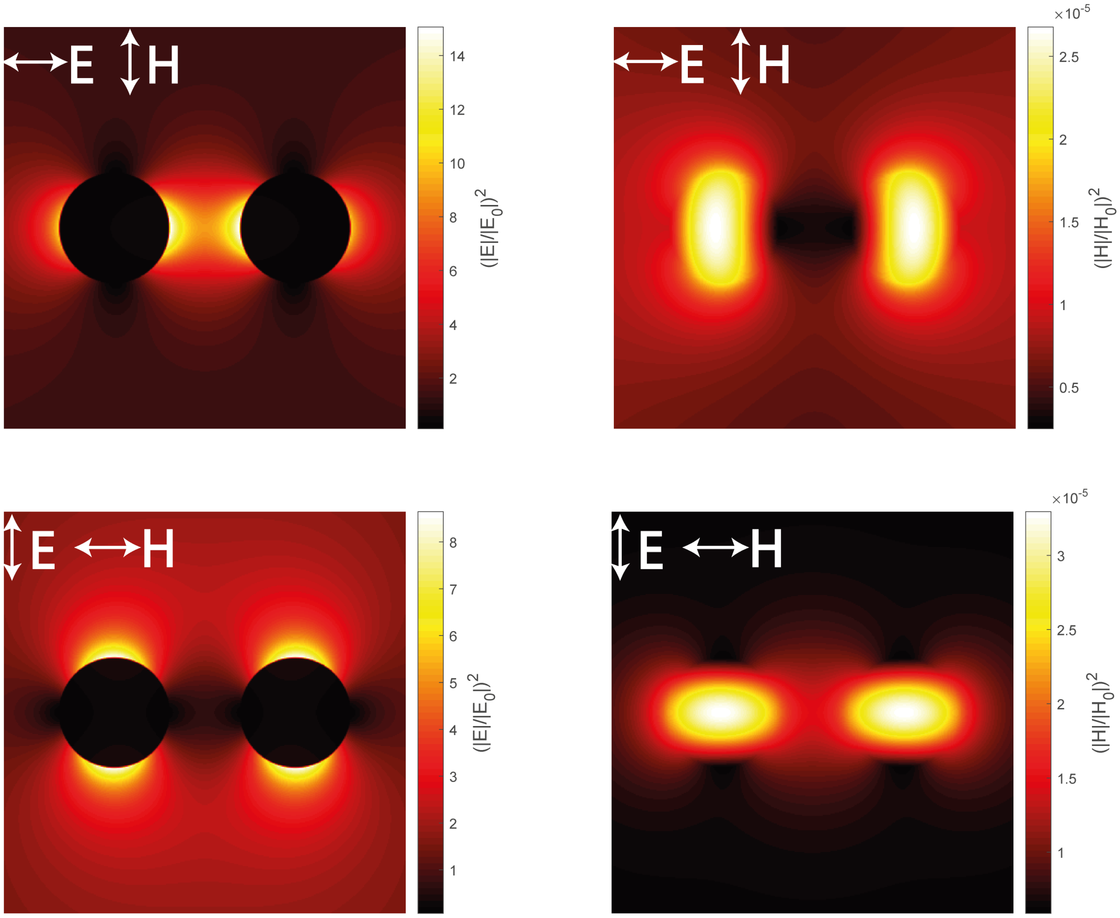

SUPPLEMENTARY FIGURE 1. Electric (E) and magnetic (H) field distributions calculated for a GaP dimer nano-antenna (height 200 nm, radius 50 nm, gap 65 nm) illuminated with a normally incident plane wave ($\lambda_{\rm exc} = 685$ nm). Relative field amplitudes are shown normalised by the amplitudes of the electric (E_0) and magnetic (H_0) fields in the incident wave. The polarisation of the incident light is shown with arrows. The distributions are calculated at half-height of the nano-antenna ($z = 100$ nm).

SUPPLEMENTARY NOTE II: LIGHT SCATTERING BY GALLIUM PHOSPHIDE DIMER NANO-ANTENNAS

The scattering cross section of a nano-particle can be defined as the ratio between the power radiated by the nano-antenna over the incident power[1] $\sigma_{\text{scatt}}(\omega) = P_{\text{rad}}(\omega)/I_{\text{exc}}(\omega)$. The corresponding absorption efficiency can be estimated as $\sigma_{\text{abs}} = \sigma_{\text{ext}} - \sigma_{\text{scatt}}$, where σ_{ext} is the extinction cross section. The relative efficiency, Q, is calculated as the ratio between the relative cross section divided by the geometric area of the dimer, given by $A_{\text{geom}} = 2 \times \pi r^2$, where r is the nano-pillar radius. Fig.2 shows the calculated scattering efficiency for different radii GaP dimer nano-antennas, and for X and Y polarization geometries. As shown, the scattering response fully overlaps with the PL emission profile of monolayer WSe_2.

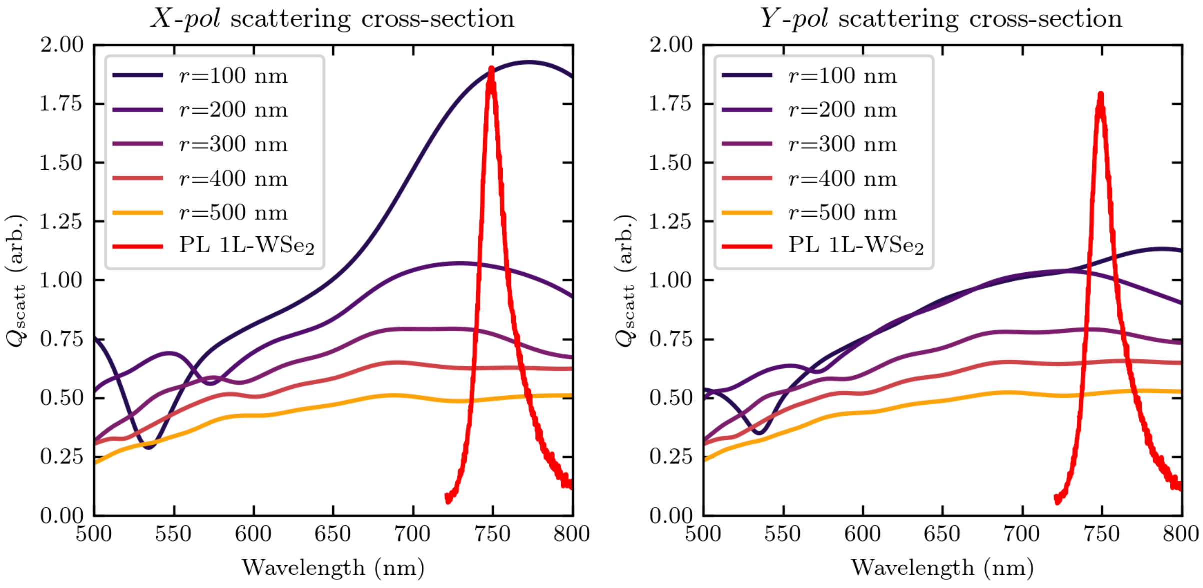

SUPPLEMENTARY FIGURE 2. Simulated scattering cross section (Q_{scatt}) for GaP dimer nano-antennas with 65 nm gap width and 200 nm height, excited at $\lambda_{\text{exc}} = 685$ nm with different polarizations, as shown in the main text in Fig.1.

SUPPLEMENTARY NOTE III: COMPARISON OF DIFFERENT IMAGING TECHNIQUES FOR WSE_2 COUPLED TO NANO-ANTENNAS

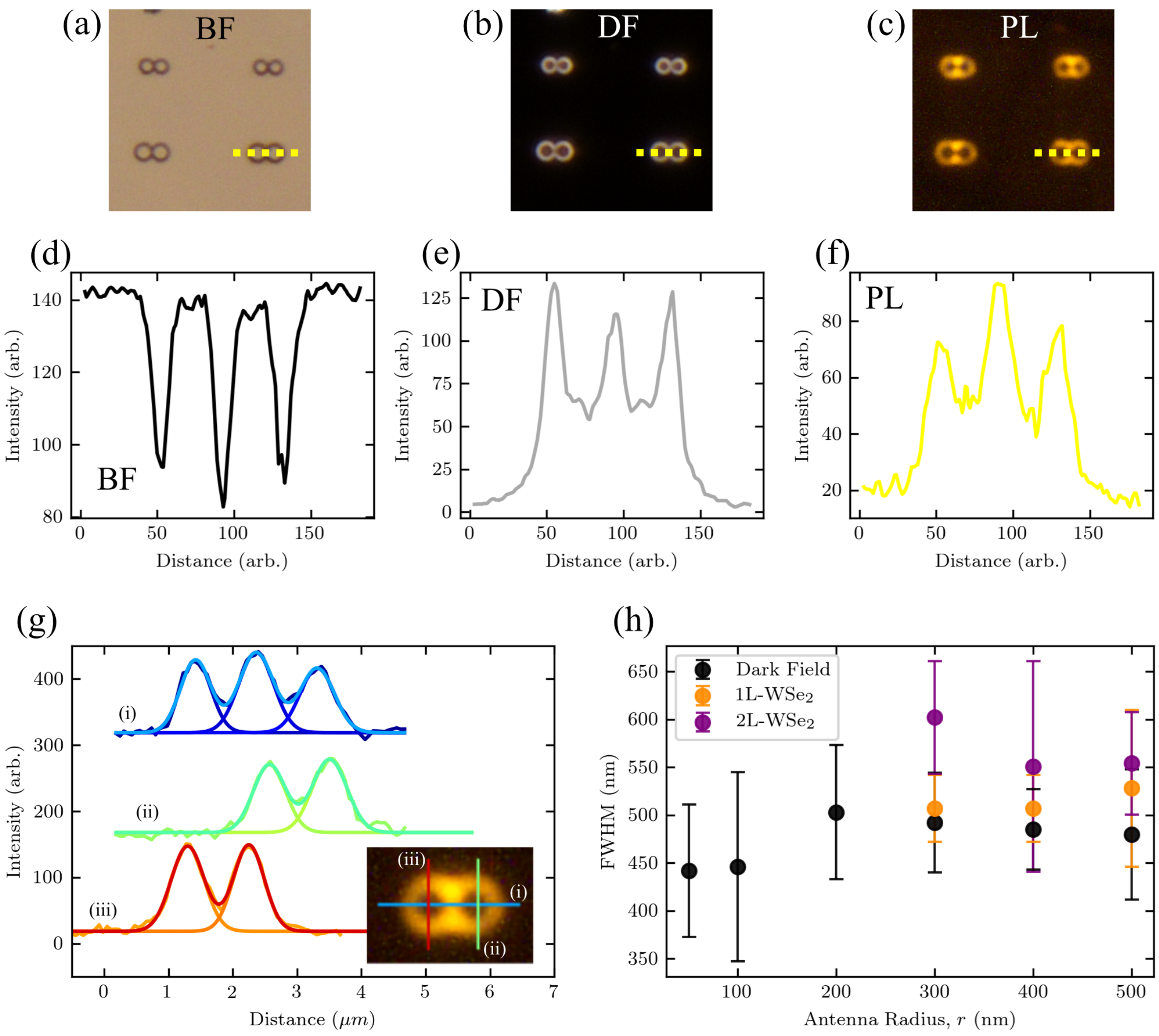

SUPPLEMENTARY FIGURE 3. (a-f) Bright-field (BF), dark-field (DF) and photoluminescence (PL) imaging with the corresponding intensity profiles (d-f) extracted from the data along the yellow dashed lines in (a-c). (g) PL imaging profiles for a single GaP dimer nano-antenna covered with 1L-WSe_2. The PL is highly localized at the nano-antenna edges, showing full-width half maximum (FWHM) values close to the optical microscope resolution. (h) FWHM of the PL and DF profiles for nano-antennas with different radii, extracted from cross-sections similar to (g). The error bars are calculated form the standard deviation of the fit values. For nano-antennas with $r < 300$ nm it is no longer possible to clearly resolve distinct peaks in the PL profiles.

SUPPLEMENTARY NOTE IV: CALCULATION OF THE PURCELL ENHANCEMENT PRODUCED BY THE GALLIUM PHOSPHIDE NANO-ANTENNAS

The Purcell enhancement is calculated as the decay rate γ_r of an emitter coupled to the GaP nano-antenna, normalised with the decay rate γ_r^0 of an emitter on a planar GaP substrate. The decay rate enhancement γ_r/γ_r^0 corresponds to the enhancement of the rate of energy dissipation P/P_0, which we calculate with a finite-difference time-domain (FDTD) method (Lumerical software). We have calculated the power P radiated by an electric dipole at 0.5 nm distance from the top surface of the GaP pillars, for different positions over the dimer, and compared that to the power P_0 radiated by the same dipole placed 0.5 nm above the surface of the planar substrate. In order to model our experimental system where photoluminescence in WSe_2 originates from the in-plane excitons, the dipole is placed parallel to the surface of the pillars or the substrate, polarised along the direction parallel or perpendicular to the dimer axis. Fig. 4a plots the 2D maps of the Purcell enhancement around the dimer, for the two dipole orientations as indicated by the white arrow. In Fig. 4b a dependence of γ_r/γ_r^0 on the position in the dimer gap is shown for a dimer with r=50 nm. Strong maxima are observed close to the edges of the pillars (marked with dotted lines in Fig. 4a) with the Purcell enhancement values reaching 15. As Fig.4c shows, the maximum Purcell enhancement values increase as r is varied from 500 to 50 nm, while their positions remain the same in the proximity of the pillar edges.

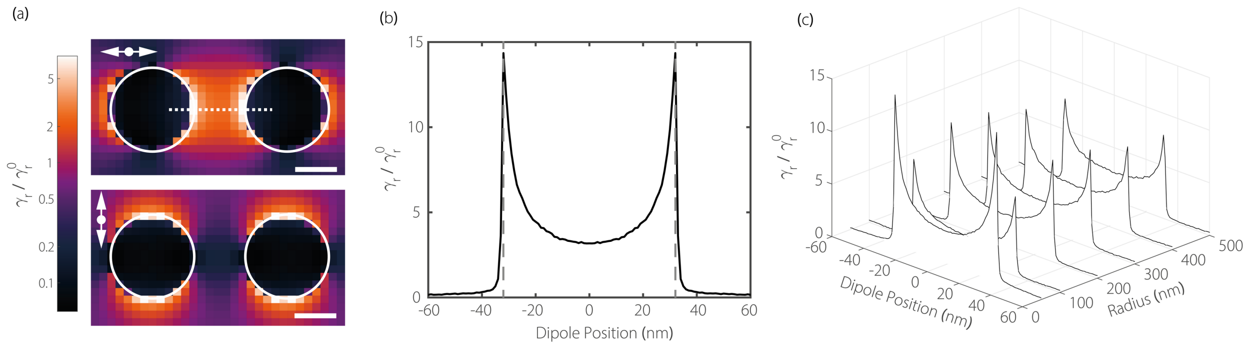

SUPPLEMENTARY FIGURE 4. (a) Maps showing decay rate enhancement γ_r/γ_r^0 for a dipolar emitter orientated parallel and perpendicular to the dimer axis, with pillars of radius r=50 nm, height h=200 nm and having a gap g=65 nm. Scale bar is 50 nm. Dependence of γ_r/γ_r^0 within the dimer gap for a dipolar emitter oriented parallel to the dimer axis shown by the dotted line in (a). The dotted lines in (b) marks the edges of the pillars. (c) Dependences of γ_r/γ_r^0 as in (b) plotted for dimers with different r, with gap fixed at 65 nm.

SUPPLEMENTARY NOTE V: CALCULATED EMISSION PATTERN FOR GALLIUM PHOSPHIDE NANO-ANTENNAS IN COMPARISON WITH PLANAR GALLIUM PHOSPHIDE

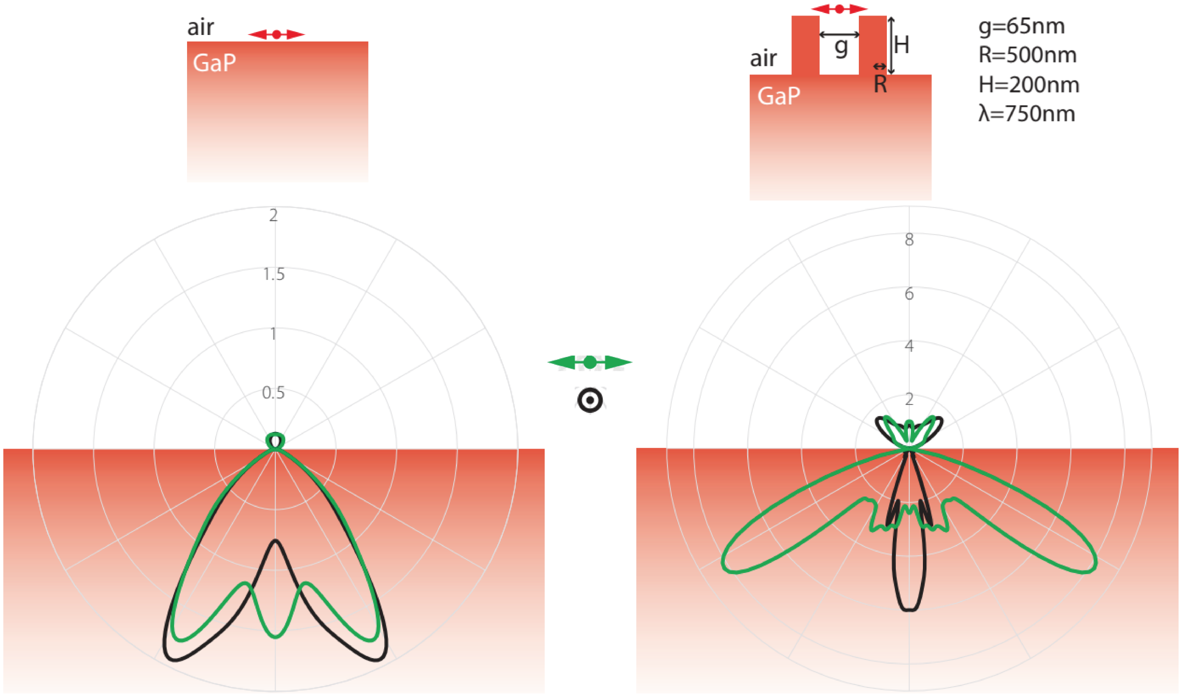

SUPPLEMENTARY FIGURE 5. Calculated emission pattern for a dipole placed on planar GaP substrate ($z = 1$nm) and one placed in the middle of the gap of a GaP dimer nano-antenna ($z = 200$nm). The emission patterns are shown for a dipole parallel (green) or perpendicular (black) to the dimer axis. Although most of the emitted light is directed into the higher index substrate, the emission in air is strongly increased due to the presence of the dimer nano-structure.

SUPPLEMENTARY NOTE VI: POLARISATION-RESOLVED PL OF WSE_2 COUPLED TO NANO-ANTENNAS

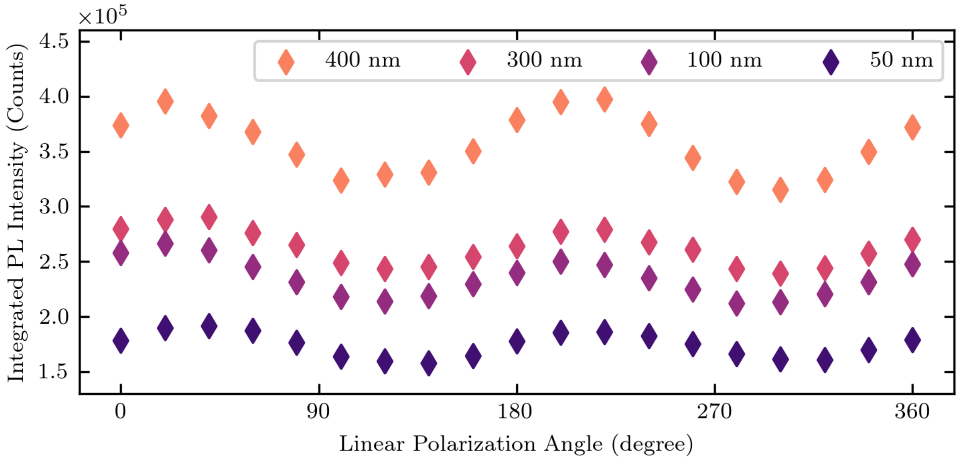

SUPPLEMENTARY FIGURE 6. WSe_2 Integrated PL intensity as a function of the angle of the linear polarisation of the excitation laser for nano-antennas of different radii and the same gap width of $g = 65$ nm.

SUPPLEMENTARY REFERENCES

[1] Novotny, L. & Hecht, B. *Principles of Nano-Optics* (Cambridge University Press, Cambridge, 2006).
